# Left ventricular simulation of cardiac compression: Hemodynamics and regional mechanics

**DOI:** 10.1371/journal.pone.0224475

**Published:** 2019-10-31

**Authors:** Edgar Aranda-Michel, Lewis K. Waldman, Dennis R. Trumble

**Affiliations:** 1 Department of Biomedical Engineering, Carnegie Mellon University, Pittsburgh, Pennsylvania, United States of America; 2 Insilicomed, La Jolla, California, United States of America; Temple University, UNITED STATES

## Abstract

Heart failure is a global epidemic. Left ventricular assist devices provide added cardiac output for severe cases but cause infection and thromboembolism. Proposed direct cardiac compression devices eliminate blood contacting surfaces, but no group has optimized the balance between hemodynamic benefit and excessive ventricular wall strains and stresses. Here, we use left ventricular simulations to apply compressions and analyze hemodynamics as well as regional wall mechanics. This axisymmetric model corresponds with current symmetric bench prototypes. At nominal pressures of 3.1 kPa applied over the epicardial compression zone, hemodynamics improved substantially. Ejection fraction changed from 17.6% at baseline to 30.3% with compression and stroke work nearly doubled. Parametric studies were conducted by increasing and decreasing applied pressures; ejection fraction, peak pressure, and stroke work increased linearly with changes in applied compression. End-systolic volume decreased substantially. Regional mechanics analysis showed principal stress increases at the endocardium, in the middle of the compression region. Principal strains remained unchanged or increased moderately with nominal compression. However, at maximum applied compression, stresses and strains increased substantially providing potential constraints on allowable compressions. These results demonstrate a framework for analysis and optimization of cardiac compression as a prelude to biventricular simulations and subsequent animal experiments.

## Introduction

Heart failure (HF) is the inability of the heart to provide enough perfusion to the body (and itself) to meet metabolic demands. This syndrome is the number one killer of Americans, contributing to one in every nine deaths in this country and afflicting about 2% (75 million) of the adult population worldwide [1–3]. The persistent five-year mortality rate of 45% in women and 65% in men [4], coupled with rising rates of coronary artery disease, high blood pressure, diabetes and obesity are likely to exacerbate the HF pandemic for the foreseeable future unless preventative measures are more widely adopted and/or new and better treatments are developed [5–10].

Studies indicate that roughly 20% of all HF cases arise from causes that cannot be prevented by lifestyle choices or treated via pharmacologic means alone[11]. Consequently, there will always be a need for permanent replacement therapies or long-term mechanical devices to support the failing heart. Currently, cardiac transplantation is considered to be the definitive treatment for end stage heart failure, but this option is limited due to the severity of the surgery, the need for lifelong medications and a small donor pool[12]. Implantable ventricular assist devices (VADs) are another alternative for patients needing long-term circulatory support, but conventional VAD therapy has its own limitations when used as “destination therapy.” VADs implanted for extended periods (months to years) have been, and continue to be, plagued by driveline infections and thromboembolic events, with prevalence as high as 12% and 17% respectively [13].

Despite serious complications associated with chronic use of these devices, VADs have the potential to meet the needs of millions of end stage HF patients if problems associated with energy transmittance and blood handling can be solved. To address this first issue, our lab has developed an implantable muscle energy converter (MEC) powered by the latissimus dorsi as a means to drive a pulsatile VAD without having to transmit energy from outside the body[14], eliminating the need for infection-prone drivelines and avoiding the complexities—and additional hardware requirements—of transcutaneous energy transmission. To solve the second problem, blood handling, we are working to develop a soft robotic sleeve compatible with the MEC actuation that can be used to increase blood flow via compression of the cardiac ventricles, avoiding blood contacting surfaces altogether.

Studies of direct cardiac compression (DCC) date back nearly 35 years but its clinical use has been limited by an inability to conduct detailed investigations to explore the mechanics of applying compression and torsional displacements to the heart with the aim of optimizing cardiac performance[15]. Among the work currently being done to develop soft robotic sleeves for cardiac compression, the most notable is Roche et al.’s use of pneumatic McKibben actuators embedded in silicone[16,17]. This latest incarnation of DCC has shown promise in augmenting cardiac output in short-term and bridge-to-transplant applications (owing to percutaneous driveline requirements) while negating the thromboembolic events due to blood contacting surfaces. However, no group has systematically examined the biomechanical effects of cardiac compression to optimize this approach. In this paper we reference compressive ventricular assist devices (cVADs) to distinguish them from our torsional assist devices (tVADs).

Analyzing the biomechanics of cardiac compression for long-term or permanent circulatory support is vital for several reasons. First, given the finite amount of energy available to muscle-powered VADs, that energy should be applied to the heart in a way that maximizes cardiac output. Second, minimization of the resulting ventricular wall stresses and strains due to cardiac compression is critical for the long-term health of the myocardium and the overall viability of the proposed system. Essentially, this approach allows for a maximization of hemodynamic improvement while ensuring that the resulting wall stresses and strains due to compression are minimized. Another potential benefit relates to cardiac remodeling, which is a difficult factor to take into account during the course of long-term cardiac support. Cardiac remodeling is predominantly responsible for the progression of ventricular hypertrophy and subsequent decompensation leading to dilation and heart failure. Cardiac compression sleeves could conceivably be used to reverse this process if the long-term effects of wall stresses imposed on the heart can be estimated and controlled. Although strains can be measured using MRI tagging or speckle tracking echocardiography, most patients are not indicated for such studies. Moreover, wall stresses can only be estimated using realistic computational modeling. Changes in hemodynamics with compression may have other potential advantages. If end-systolic volumes can be decreased with cardiac compression, there may be added potential to promote reverse remodeling of severely dilated hearts. Exploiting these simulation methods allows us to estimate both global hemodynamics and regional wall mechanics and to strike a balance between hemodynamic benefit and excessive wall strains and stresses.

Optimizing cardiac compressions to reduce the resulting wall strains and stresses to levels found in healthy hearts would reduce the chance of deleterious cardiac remodeling over the long term.[14] In this paper we describe a framework for parametric studies of compression on a prolate spheroidal model of the left ventricle utilizing a multiscale cardiac finite element analysis software package, *Continuity Pro* (Insilicomed, Inc., La Jolla, CA). The software is a derivative work of the Continuity software platform developed by the Cardiac Mechanics Research Group in the Bioengineering Department of UCSD. Our group has used it previously to explore the effects of applied apical torsion on both single ventricle and biventricular models of the heart[18,19]. The hemodynamics and regional mechanics results from compression simulations are analyzed to draw broad inferences about the efficacy of cardiac compression. And, they are designed to establish a functional framework for cardiac compression simulations that can then be correlated with animal experiments and applied to patient-specific biventricular HF models.

## Materials and methods

### Computational models of heart failure

These computational models combine high-order finite element analysis, myofiber architecture, a nonlinear constitutive law, and a dynamic model of myocardial excitation-contraction coupling. They are coupled to lumped-parameter closed-loop circulatory models that allow simulations to achieve steady-state cardiac cycles. This allows for correlation of models with clinical data from patients to obtain realistic simulations of heart failure and many of its comorbidities. The models developed here were based on the same mid-range biventricular heart failure model, chosen from more than 20 available models, used in previous work on ventricular torsion (named torsion ventricular assist device or tVAD)[19]. Since that patient cohort involved dyssynchronous heart failure (average ejection fraction of 27%) and treatment with cardiac resynchronization therapy, cardiac contraction was reduced to simulate severe heart failure more appropriate for VAD.

**Fig 1:**
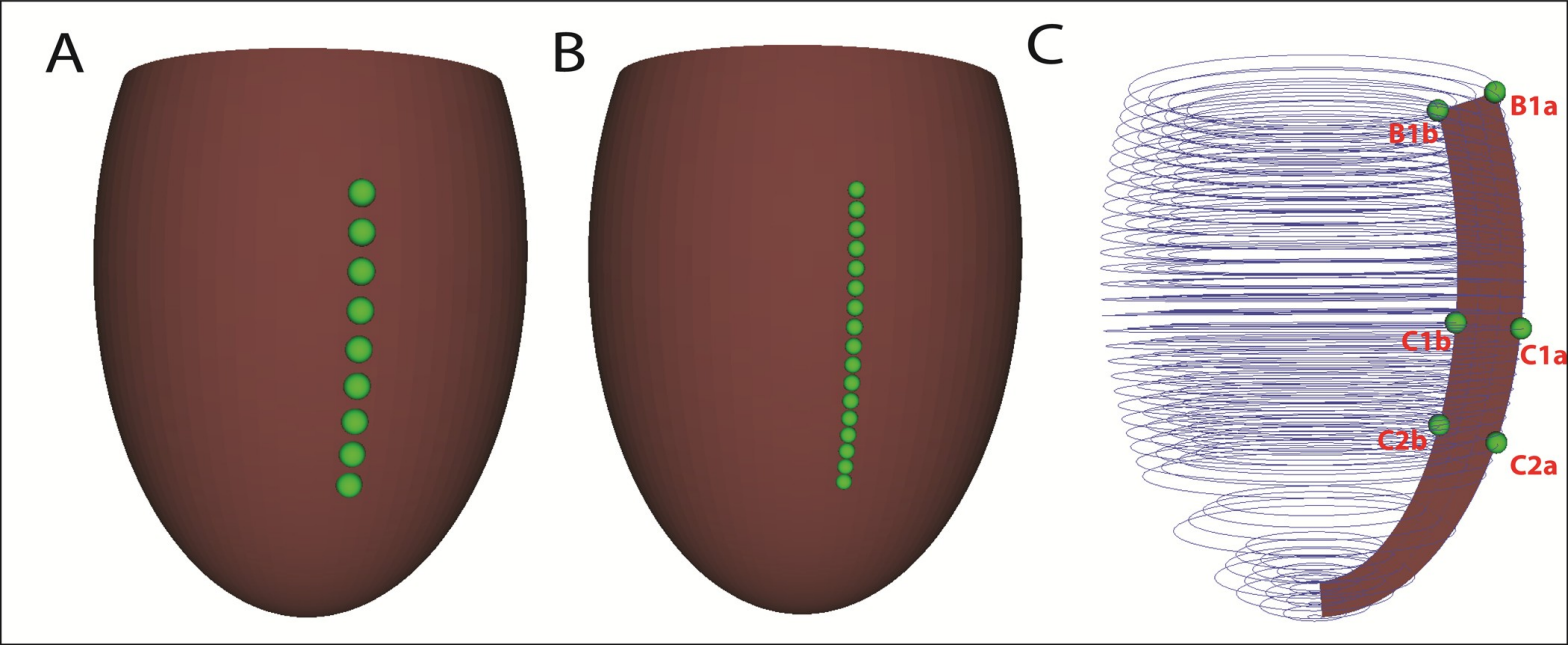
Finite element convergence models. (A) Epicardial rendering of model with 9 epicardial nodes for applied force application in no load state. (B) Epicardial rendering of refined model with 17 epicardial nodes for applied force application in no load state. (C) Cross-sectional rendering of the finite elements for the 17-node model showing key nodes of the axisymmetric model at which ventricular wall strains and stresses are calculated. The top two nodes are the epicardial (B1a) and endocardial (B1b) nodes at the ventricular base. The center nodes are in the middle of the compression region and are designated C1a (epicardium) and C1b (endocardium. The lowest nodes are at the apical end of the compression zone and are designated C2a (epicardium) and C2b (endocardium).

To analyze how the initial design of a circular compressive sleeve (compressive ventricular assist device or cVAD) would interact with the heart, two axisymmetric left ventricular models were constructed in prolate spheroidal coordinates **(Fig 1)**. Both models are axisymmetric and have three layers with cubic Hermite interpolation transmurally. One has 48 elements with cubic Hermite interpolation longitudinally and 9 nodes for force application (**Fig 1A**). The other model is refined once longitudinally, resulting in 96 elements, and 17 nodes, along the same length, for force application and has linear interpolation in that direction (**Fig 1B**). The models have linear interpolation circumferentially due to axisymmetry. These models were employed to perform convergence studies in order to elucidate the degree of longitudinal resolution needed to obtain accurate results in preparation for large-scale biventricular modeling. The cross-section of the larger model shows three layers of finite elements transmurally (**Fig 1C**).

There are two coordinates systems employed in the analysis. The first are the global coordinates, λ, μ, and ϑ, corresponding to the transmural, longitudinal and circumferential directions. The second are the fiber coordinates, which are in the fiber, cross-fiber, and transmural directions respectively. The fiber orientation changes from -37 degrees to 83 degrees between the epicardium and the endocardium and is uniform, i.e., no variation from one ventricular site to another. The global coordinates provide the capability to utilize the λ coordinate for cardiac compression, just as the ϑ coordinate was used in previous work on ventricular torsion[19]. The boundary conditions are fixation of all basal nodes in μ to simulate the valve plane, and fixation of the epicardial basal node in the other directions. The constitutive law was identical to that used previously[19], slightly compressible transverse isotropy in which the fiber axes are considerably stiffer than the transverse plane. The bulk modulus is set high enough so that the finite elements always remain isochoric. The dynamic model was also identical to that used previously. In the earlier biventricular model dual closed-loop, lumped parameter circulatory models were used. These were reduced to a single closed-loop circuit. Circulatory model parameters were modified to effect severe heart failure and are provided in **Table 1**. The simplified LV circulatory system includes an arterial and venous vessel, each with a volume, compliance, and resistance. The models have identical no-load cavity volumes (135 ml) when compared to the left ventricle of the biventricular model used for torsion studies[19]. Although the models have idealized geometry to match initial sleeve designs, all other attributes are either identical or, in the case of the circulatory model, similar to the left ventricle of the more advanced biventricular models.

**Table 1:** Lumped parameter circulatory values. These values are from the baseline HF model at ED of a steady state cardiac cycle and are the initial conditions for. all compression simulations.

Circulatory Parameters	
**Initial Conditions**	
**Arterial Volume**	**555.46 *ml***
**Venous Volume**	**2296.22 *ml***
**Fixed Parameters**	
**Arterial Vessel**	
Compliance	**55 $\frac{ml}{kPa}$**
Resistance	**100 $\frac{kPa*ms}{ml}$**
Impedence	**5 $\frac{kPa*ms}{ml}$**
**Venous Vessel**	
Compliance	**433 $\frac{ml}{kPa}$**
Resistance	**50 $\frac{kPa*ms}{ml}$**

**Fig 2:**
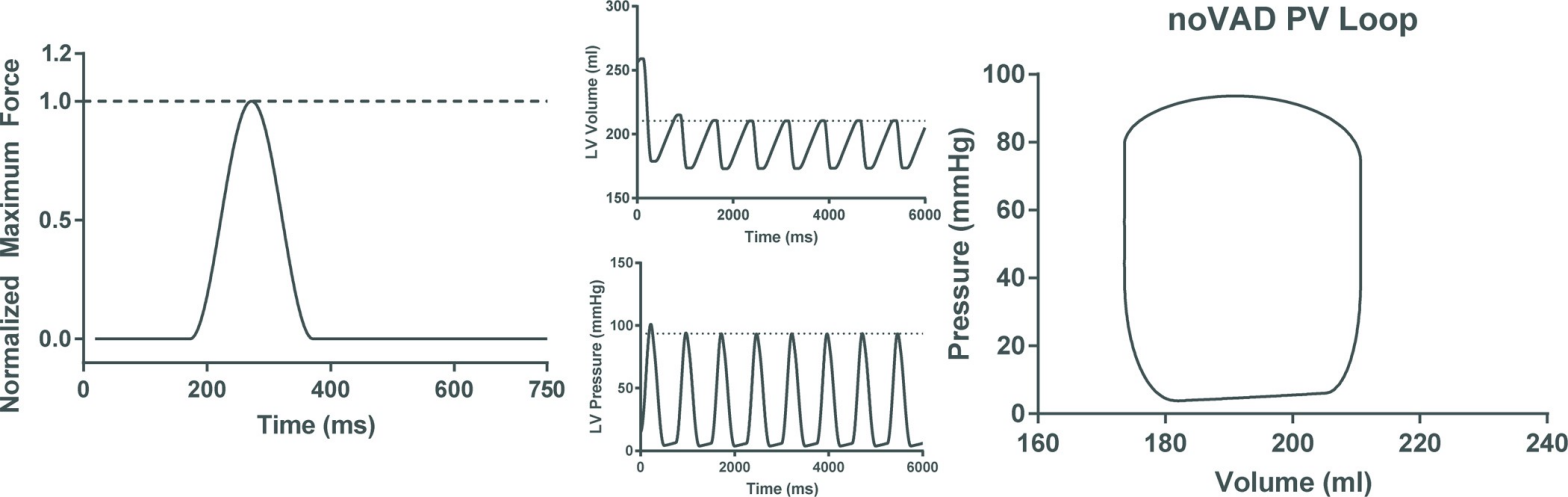
Left ventricular heart failure model. (A) Applied force at all force application nodes as a function of time employed to compress LV throughout the simulation cardiac cycle of 750ms. Applied forces are zero throughout isovolumic contraction. Force application commences at 154 ms, peaks at 254 ms, and decreases until it returns to zero at 354 ms and remains there for the remainder of the cardiac cycle during filling. The maximum applied force is scaled by the peak value of the sinusoid so that only at 254 ms will the maximum force be applied. Time tracings of (B) left ventricular volume and (C) left ventricular pressure showing rapid equilibration of the simulation. (D)The pressure-volume loop for the baseline heart failure model after equilibration (last cardiac cycle shown in (B) and (C)).

All simulations, baseline heart failure (no ventricular assist device, or noVAD) and cardiac compression (compressive ventricular assist device or cVAD) were run with the cardiac cycle lasting 750 ms. The initial end diastolic pressure of the baseline model was set at 2 kPa. To simulate compression, a sinusoidal force profile is applied on the nodes described earlier of both models in the λ direction inwards toward the endocardium (**Fig 2A**). This force profile was designed to avoid applying forces during isovolumic contraction, start applying them gradually at the beginning of ejection (154 ms), continue to a peak compression near end-ejection just after peak pressure (254 ms), plateau, and fall off to zero near the end of isovolumic relaxation (354 ms) in every cardiac cycle. Following earlier characterizations of cardiac compression *in-vivo*, a constant force distribution peaking at 7.5 N (nominal compression) was applied over the compression nodes of the LV models [20].

Baseline HF simulations were run for 8 cardiac cycles (**Fig 2B, 2C and 2D**). Stability and equilibration were assessed by plotting time series of the left ventricular pressure and volume **(Fig 2B and 2C).** The corresponding pressure-volume loop from the final cardiac cycle is shown in **Fig 2D**. Hemodynamics were virtually identical for both models. The compression simulations have initial conditions that are the same as those at end-diastole of the final cycle of baseline simulations.

A convergence test was performed to compare the regional mechanics of the 9 and 17 compression node models in preparation for future research involving large biventricular models in which simulation runtimes will be much longer and obtaining accurate results while minimizing the size of models will be more efficient and allow for parametric studies. A nominal peak force of 7.5 N was applied at each of the 9 or 17 nodes in the compression region of the models–both spanning the same epicardial surface area. After running the compression models with the nominal peak forces applied and using the final cardiac cycle after equilibration for post-processing, the peak forces were raised and lowered in increments of 25% to analyze how hemodynamics change with alterations in the applied forces. Myocardial thickening for nominal compression at the center of the zone was studied to estimate changes that occur with cardiac compression. Lastly, regional mechanics for nominal compression were analyzed in terms of maximum and minimum principal strain and stress distributions at peak compression. The component stresses and strains in fiber coordinates that contribute most to the principal values were plotted as a function of time in the cardiac cycle to study how the fiber architecture contributes to principal values.

It is useful to estimate applied epicardial pressures equivalent to the applied forces for device development and generalization to various sized hearts. These forces can be converted to an equivalent pressure by dividing them by the area between any two circumferences in the compression zone at a given time during the cardiac cycle. The area is an average of circumferences between any two compression nodes multiplied by the longitudinal distance between the circumferences. Since there is a small variation in these areas as a function of longitude, a small variation in equivalent pressures occurs despite uniform force application. For this nominal compression case, the average pressure applied to the LV in the 16 rings was 3.1 kPa at peak compression with a range of 2.7 to 3.3 kPa.

The simulated regional mechanics are presented in terms of finite strains and corresponding Cauchy stresses (kPa) at certain finite element nodes in the model. And, they are shown as color-coded renderings varying over epicardial and endocardial surfaces at certain times in the cardiac cycle. Principal strains are the three eigenvalues of the symmetric Lagrangian strain tensor referred to the no load state. Principal stresses are the eigenvalues of the Cauchy stress tensor. We focus on maximum and minimum principal strains and stresses. The strain and stress tensors are the results of our three-dimensional finite elasticity analysis in which the large deformation mechanics of a transverse isotropic material is analyzed without any assumptions from membrane or shell theory, as in our earlier work[19]. The symmetric tensors are referred to the fiber architecture in the fiber, cross-fiber and transmural directions. Hence, the tensors are estimated in terms of normal and shear components (1 is the fiber direction, 2 is the cross-fiber direction and 3 is the transmural direction).

## Results

### Convergence test

**Fig 3:**
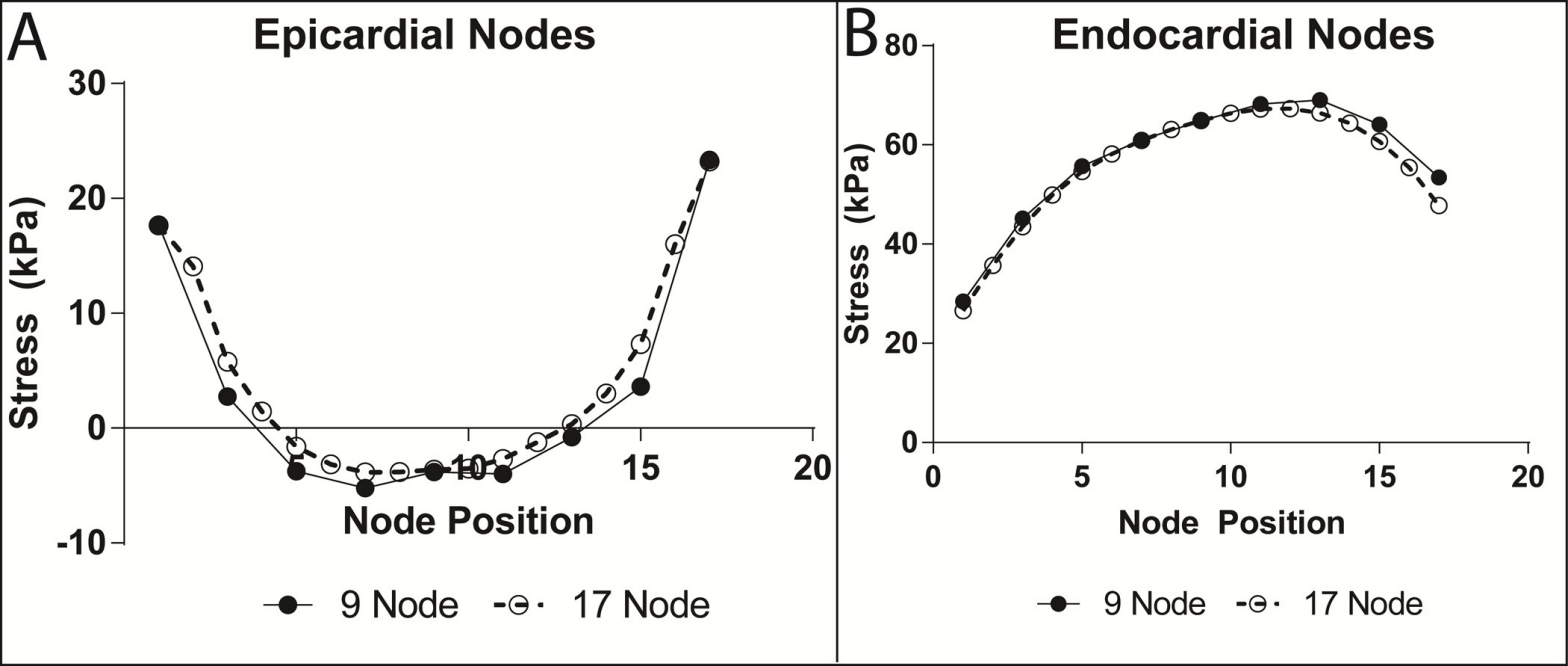
Convergence test for longitudinal refinement. (A) Epicardial maximum principal stress plotted at peak compression for both models at applied force nodes. (B) Endocardial maximum principal stress plotted at peak compression for both models at applied force nodes. Note close correspondence of epicardial stresses with small underestimation of stresses using the 9-node model. Also, note very close match of larger endocardial stresses with small overestimation of values by the 9-node model toward the apical part of the compression zone.

Due to axisymmetry and cubic interpolation transmurally, further refinement circumferentially or transmurally is unnecessary to study convergence properties of the models. Therefore, the model was only refined longitudinally (in μ) and tested for convergence, and not in λ or ϑ. The maximum principal stresses in the compression region were analyzed for both models at the epicardium and endocardium **(Fig 3A and 3B)** and compared; since local stresses are inherently less accurate than strains or hemodynamics, this comparison is a good test. Due to the similarity of the stress results, the 9-node (smaller) model would be sufficient for simulations. However, in the future when we progress to a biventricular model, the size and complexity of the simulations will greatly increase to incorporate the right ventricle (RV), add the pulmonary circulation and apply non-uniform forces around the circumferences to avoid excessive RV compression. Being able to reduce the number of nodes and elements in any direction will make much larger simulations viable. Although the 9-node (smaller) model is sufficiently accurate, the larger model with 17 nodes and circumferences equilibrates rapidly with this idealized geometry and was used in the remainder of this study.

### Nominal compression

**Fig 4:**
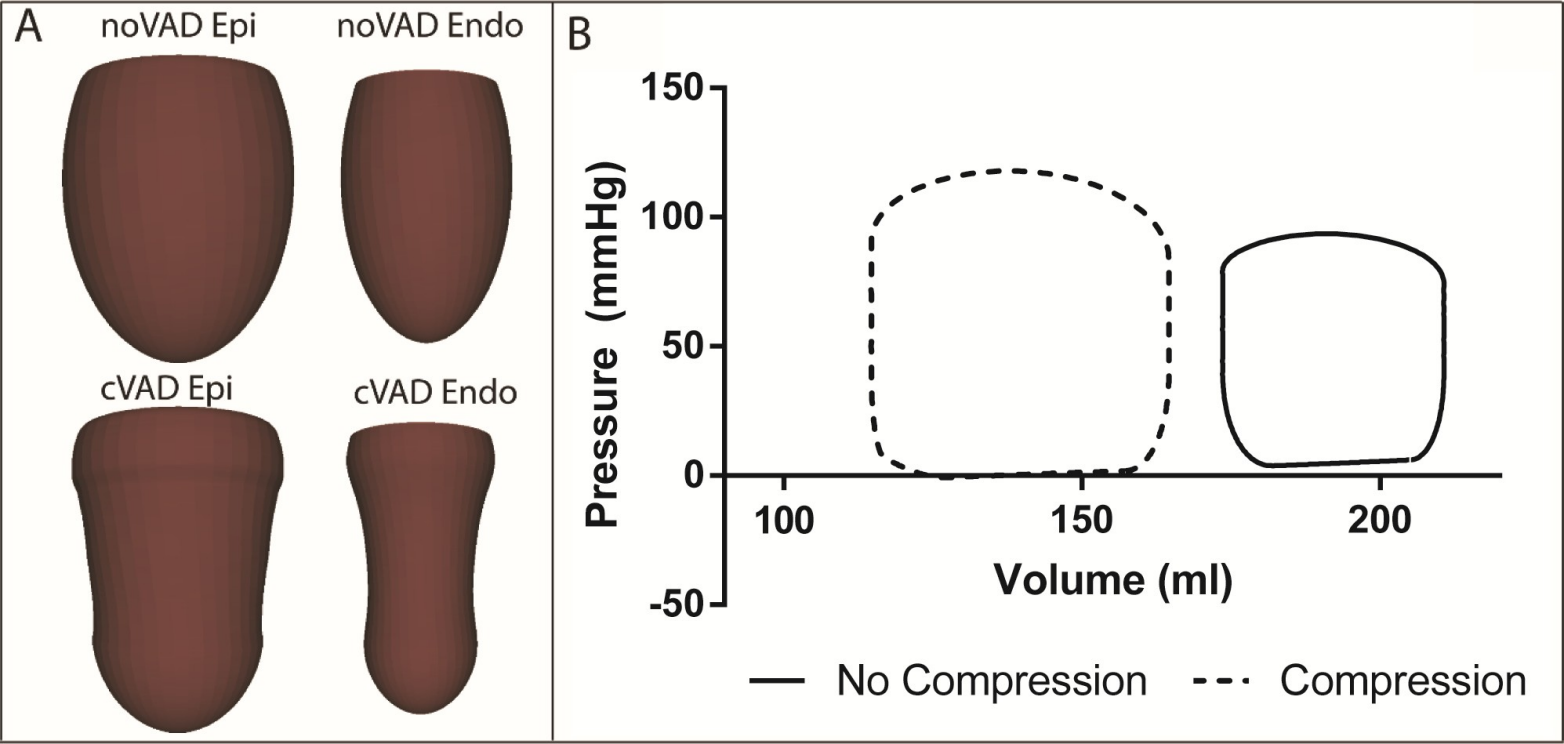
Nominal compression global hemodynamics. (A) Epicardial and endocardial surface renderings of baseline HF and the nominal compression simulation near end-ejection when peak compression of cVAD model occurs. (B) Pressure-Volume loops for the converged solutions of the baseline heart failure and nominal compression simulations. Note the large increase in the size of the P-V loop with compression and its complete separation from the baseline HF loop.

The epicardial surface, on which the forces are applied in the compression simulations (cVAD), shows substantial indentation at peak compression when compared with baseline HF **(Fig 4A)**. The corresponding endocardial surface is also highly deformed relative to the baseline endocardial geometry at that time in the cardiac cycle. The pressure-volume (PV) loops show a large improvement in hemodynamics resulting from the applied forces. Stroke work (SW) increased from 0.42 J to 0.74J, peak pressure increased from 93.6 mmHg to 117.9 mmHg and ejection fraction (EF) increased from 17.6% to 30.3% **(Fig 4B)** in the nominal compression simulation.

### Parametric force application

**Fig 5:**
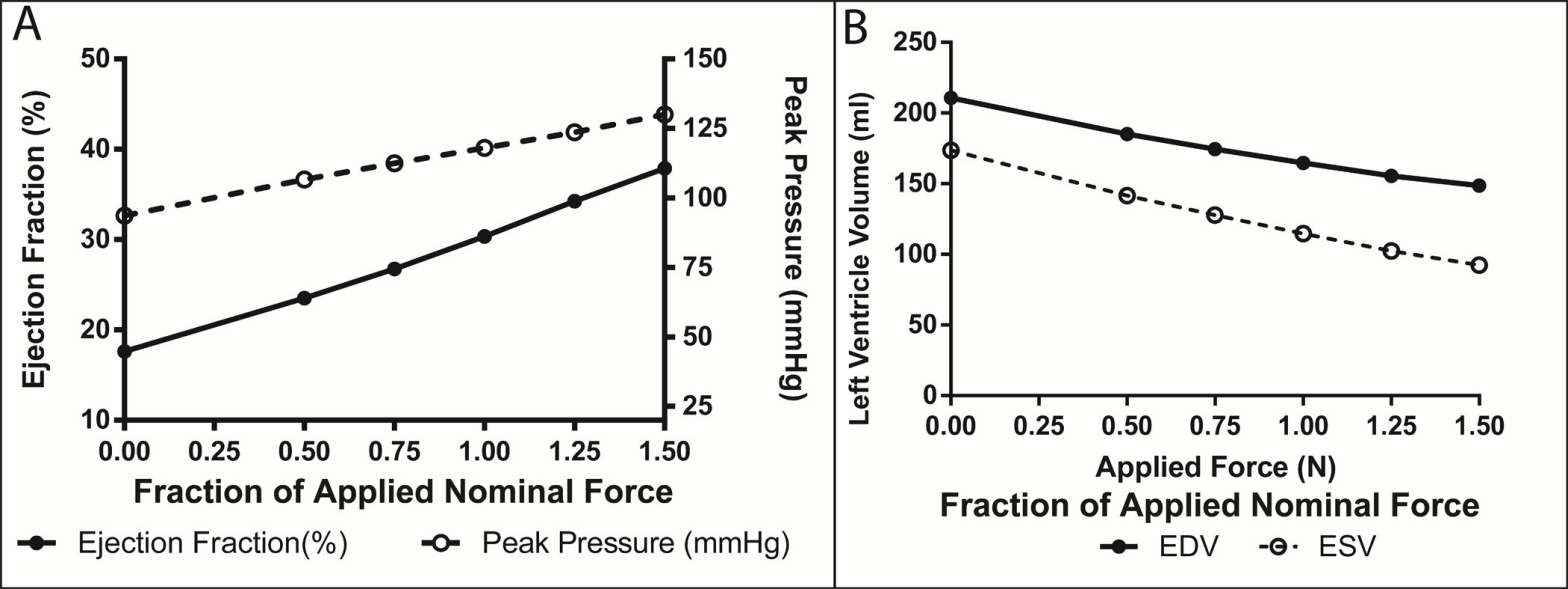
Hemodynamic variations as applied forces change magnitudes. (A) Changes in ejection fraction and peak pressure as functions of applied forces. (B) Changes in end-diastolic and end-systolic volume as functions of applied forces. Note: Value of 1.0 on the abscissas indicates nominal forces at peak compression.

The baseline HF simulation has a stroke volume of 37.1 ml, EF of 17.6%, peak pressure of 93.6 mmHg, and SW of 0.42 joules (**Fig 5 and Table 2)**. Nominal compression (NC) shows an increase in most hemodynamic parameters with a stroke volume of 50.0 ml, EF of 30.3%, peak pressure of 117.9 mmHg, and SW of 0.72 joules (**Fig 5 and Table 2)**. There is a predominantly linear dependence between the hemodynamic output and applied forces used (**Fig 5A and 5B)**. EF ranges from 23.5% to 37.9% and SW ranges from 0.55 joules to 0.91 joules as applied forces are raised and lowered from the nominal forces (**Fig 5 and Table 2)**. Similarly, peak pressure increases from 106.6 to 130.0 mmHg over the same force range **(Fig 5A)**. This range is equivalent to a change from 3.75 N to 11.25 N **(Fig 5A)**. The end-systolic and end-diastolic volumes decrease fairly linearly but diverge as increasing forces are applied

**Table 2:** Global hemodynamic changes. These values are from the baseline HF case, the nominal compression, and the parametric variation of the applied forces. Headings reflect fraction of nominal compression forces used in each compression simulation; note: these correspond with the plots shown in Fig 5 and the strain and stress values shown in Table 3.

Global Hemodynamic Changes						
	HF	0.5NC	0.75NC	NC	1.25NC	1.5NC
*Stroke Volume*	37.1 ml	43.5 ml	46.6 ml	50.0 ml	53.3 ml	56.3 ml
*Ejection Fraction*	17.6%	23.5%	26.7%	30.3%	34.4%	37.9%
*Peak Pressure*	93.6 mmHg	106.6 mmHg	112.5 mmHg	117.9 mmHg	123.7 mmHg	130.0 mmHg
*Stroke Work*	0.42 joules	0.55 joules	0.63 joules	0.72 joules	0.81 joules	0.91 joules
HF–Heart Failure Case, NC—Nominal Compression Case (3.1 kPa)						

### Myocardial thickening

**Fig 6:**
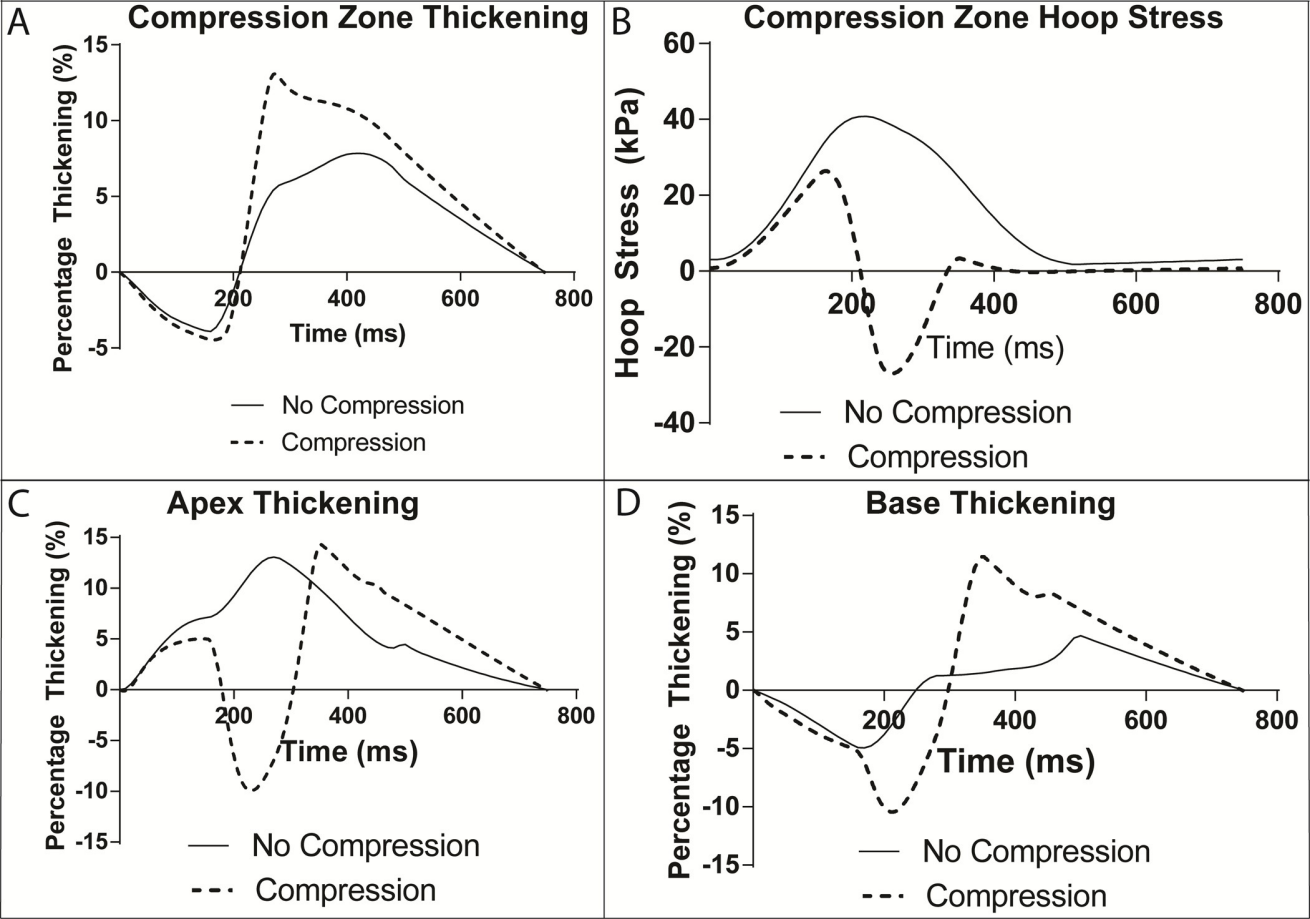
Analysis of myocardial thickening. Thickening percentage throughout the cardiac cycle for baseline HF (no compression) and compression simulations in the middle of the compression zone (A) and the corresponding hoop stress (B). Corresponding thickening at the apex (C) and the base of the heart (D). This is the percentage change in the entire myocardial wall thickness compared with the end diastolic wall thickness.

The change in myocardial thickness is presented as the percent thickening of the ventricular wall in the middle of the compression zone as a function of time in the cardiac cycle **(Fig 6A).** The corresponding hoop or circumferential stress at that location is also shown (**Fig 6B**). Thickening at the ventricular base **(Fig 6C)**, and apex **(Fig 6D)** are also provided to show how thickening changes at baseline and during compression when longitudinal location is changed. In the compression zone, the percent thickening at baseline and during compression peak at different times, with the maximum compression values occurring at the same time as peak compression and baseline values peaking later in the cardiac cycle. The baseline HF simulation has a maximum thickening of 8% whereas thickening reaches 13% during compression. Baseline thickening at the apex changes to wall thinning at peak compression. But it returns to substantial thickening later in the cardiac cycle. Baseline thickening at the base is small throughout the cardiac cycle. Like the apex, the wall is thinning at peak compression with a delayed return to thickening greater than normal late in the cardiac cycle. Fiber stress at a transmural depth where fibers are circumferential was examined because fiber stress is the same as circumferential or hoop stress at this depth **(Fig 6B)**. At baseline, the hoop stress is positive (tensile) and monotonic peaking at about 40 kPa. There is a large decrease in hoop stress with compression. It changes sign to a compressive stress nearing -28 kPa at peak compression.

### Principal and component ventricular wall strains and stresses

**Fig 7:**
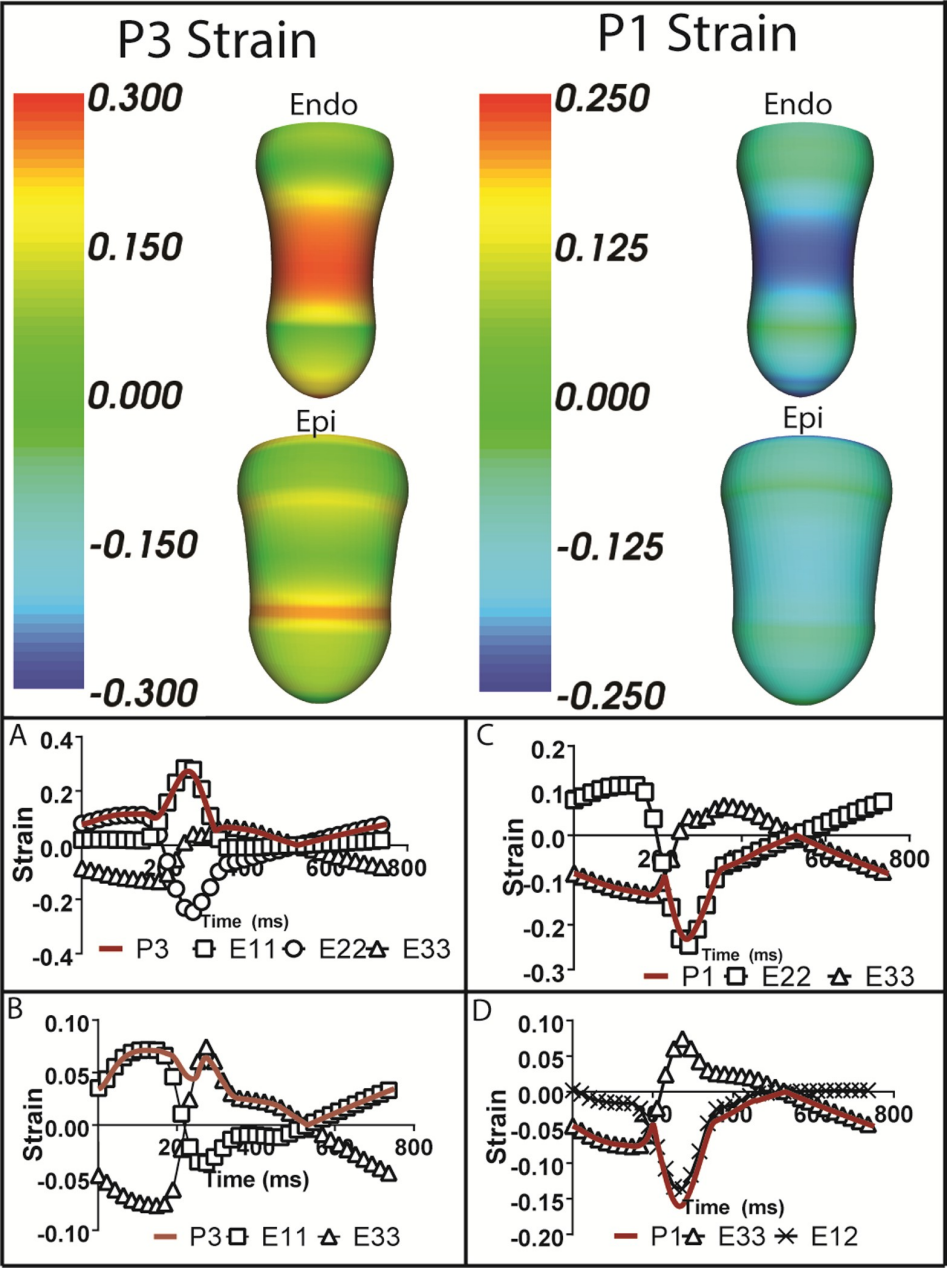
Principal strain rendering and time plots of principal and key contributing strain components in fiber coordinates. (Top) Maximum and minimum principal strain renderings on the epicardial and endocardial surfaces at nominal peak compression. (A and B) Time series of maximum endocardial and epicardial principal strain and main contributing strain components. (C and D) Time series of minimum endocardial and epicardial principal strain and main contributing strain components. Note: All time renderings come from the middle of the compression. P3 is the maximum principal strain. P1 is the minimum principal strain. Fiber coordinates 1, 2 and 3 correspond to fiber, cross-fiber and transmural directions, respectively, i.e., E11, E22 and E33 are the normal stresses.

As shown in **Fig 7 (Top)** and **Table 3**, in the endocardium the maximum principal strain is distributed over the endocardium beneath the compression zone at maximum loading during nominal compression (see Methods), while the minimum principal strain is more concentrated toward the middle of the zone; both fall off rapidly toward the base and apex. In the epicardium, the maximum principal strain is concentrated on the boundaries of the compression zone, particularly closer to the apex. The minimum principal strain is small and is distributed diffusely throughout the epicardium, with reductions around the boundary of the compression zone. Specific values for principal strains and stresses at key nodes (**Fig 1**) are provided in **Table 3** for the baseline HF case and the parametric studies of peak loading above and below nominal compression (NC). The maximum principal strain at node B1a shows virtually no change from baseline to NC and a modest increase at 1.5 NC (0.21 to 0.26). Values at node B1b are nonmonotonic with changes in peak loading; they decrease from baseline (0.25) to NC (0.16) and rise back at 1.5 NC to 0.27. Maximum strain at node C1a for the HF case is 0.14. At NC, this value decreases to 0.07. However, at 1.5 NC, it increases to 0.22. Maximum strains at node C1b are virtually unchanged from HF to NC, but they go up substantially at 1.5 NC (0.26 to 0.38). Maximum strain at node C2a steadily increases as maximum loading increases to a value of 0.41 at 1.5 NC. This value is the largest principal strain estimated in these studies (see Discussion). Strains at node C2b are nonmonotonic with changes in peak loading; they fall from a value of 0.26 at baseline to low values and rise to a substantial value, 0.32, at 1.5 NC. The minimum principal strain at node B1a is -0.21 in the HF case; this remains virtually unchanged at NC. However, the strain increases to moderate levels at 1.5 NC (-0.26), i.e. it becomes more negative. The minimum strain at B1b decreases as maximum load increases, leveling off at -0.14 for the 1.25 NC and 1.5 NC cases. The minimum strain at node C1a is small and similar for the HF and NC cases, and it increases to -0.23 at 1.5 NC. Similarly, the values at C1b are identical for the HF and NC cases (-0.23). This value increases to -0.34 at 1.5 NC. This is the largest compressive strain estimated in these studies (see Discussion). The minimum strain at C2a remains small for the different loading cases, and it increases to -0.19 at 1.5 NC. The minimum strain at node C2b is nonmonotonic with increasing maximum load. It has a value of -0.23 in the HF case, decreases to -0.15 at NC, and returns to -0.22 at 1.5 NC.

**Table 3:** Tabluation of maximum and minimum principal stress and strain in parametric analysis. Comparison of regional wall mechanics between the baseline heart failure case, the nominal compression case and the parametric variation of applied forces corresponding with Table 2 and Fig 5. The maxmimum and minimal principal stresses and strains at the nodes indicated in Fig 1C are shown. B1a and B1b are the epicardial and endocardial nodes at the ventricular base; C1a and C1b are epicardial and endocardial nodes near the center of the compression zone; C2a and C2b are epicardial and endocardial nodes at the apical end of the compression zone.

	**Maximum Principal Strain (E)**							**Maximum Principal Cauchy Stress (T kPa)**					
Node	***HF***	***0*.*5NC***	***0*.*75NC***	***NC***	***1*.*25NC***	***1*.*5NC***		***HF***	***0*.*5NC***	***0*.*75NC***	***NC***	***1*.*25NC***	***1*.*5 NC***
**B1a**	0.21	0.18	0.19	0.20	0.23	0.26	**B1a**	1.61	6.38	11.1	15.4	19.2	21.8
**B1b**	0.25	0.20	0.18	0.16	0.16	0.27	**B1b**	46.2	39.6	35.9	32.2	28.3	24.9
**C1a**	0.14	0.10	0.08	0.07	0.15	0.22	**C1a**	45.8	38.1	34.9	32.2	29.6	27.7
**C1b**	0.26	0.18	0.20	0.27	0.35	0.38	**C1b**	29.7	39.6	49.4	65.7	103.4	145.0
**C2a**	0.13	0.11	0.16	0.24	0.33	0.41	**C2a**	44.2	38.0	35.0	32.1	29.5	28.4
**C2b**	0.26	0.18	0.14	0.18	0.24	0.32	**C2b**	29.9	35.9	39.3	44.5	53.1	65.7
	**Minimum Principal Strain (E)**							**Minimum Principal Cauchy Stress (T kPa)**					
Node	***HF***	***0*.*5NC***	***0*.*75NC***	***NC***	***1*.*25NC***	***1*.*5 NC***	Node	***HF***	***0*.*5NC***	***0*.*75NC***	***NC***	***1*.*25NC***	***1*.*5 NC***
**B1a**	-0.21	-0.20	-0.21	-0.22	-0.24	-0.26	**B1a**	-38.6	-37.7	-41.3	-46.0	-53.3	-60.1
**B1b**	-0.22	-0.19	-0.17	-0.15	-0.14	-0.14	**B1b**	-12.7	-14.1	-14.9	-15.6	-16.4	-17.2
**C1a**	-0.15	-0.11	-0.12	-0.16	-0.21	-0.23	**C1a**	-0.35	-8.52	-16.8	-25.2	-37.2	-45.2
**C1b**	-0.23	-0.18	-0.16	-0.23	-0.30	-0.34	**C1b**	-12.6	-14.3	-19.4	-26.8	-39.2	-56.4
**C2a**	-0.14	-0.11	-0.11	-0.13	-0.16	-0.19	**C2a**	-0.39	-10.2	-14.3	-18.0	-19.7	-21.9
**C2b**	-0.23	-0.18	-0.16	-0.15	-0.18	-0.22	**C2b**	-12.6	-14.3	-15.2	-17.6	-21.8	-25.7

Time profiles of the maximum and minimum principal strains in the endocardium and epicardium as well as their major contributing components in fiber coordinates are provided to show how they develop during the cardiac cycle and how they relate to the underlying fiber architecture. In the endocardial time tracing of maximum principal strain **(Fig 7A)**, the strain is initially dominated by the cross-fiber strain, however, this quickly changes to the fiber strain, and not until much later in the cardiac cycle does the cross-fiber strain again dominate. The minimum principal epicardial strain **(Fig 7B)** has an opposite dynamic, changing from fiber strain to a transmural strain and back to fiber strain. The minimum principal endocardial strain **(Fig 7C)** is initially dominated by the transmural strain until the cross-fiber strain takes over. This dominates for much of the cardiac cycle until the transmural strain again dominates. Interestingly, the minimal principal epicardial strain **(Fig 7D)** is the only principal strain to have a major component of shear. The principal strain is initially dominated by transmural strain; this changes to fiber/cross-fiber shear strain and finally changes back to transmural strain. There are no significant contributions of fiber strains to the minimum principal strains.

**Fig 8:**
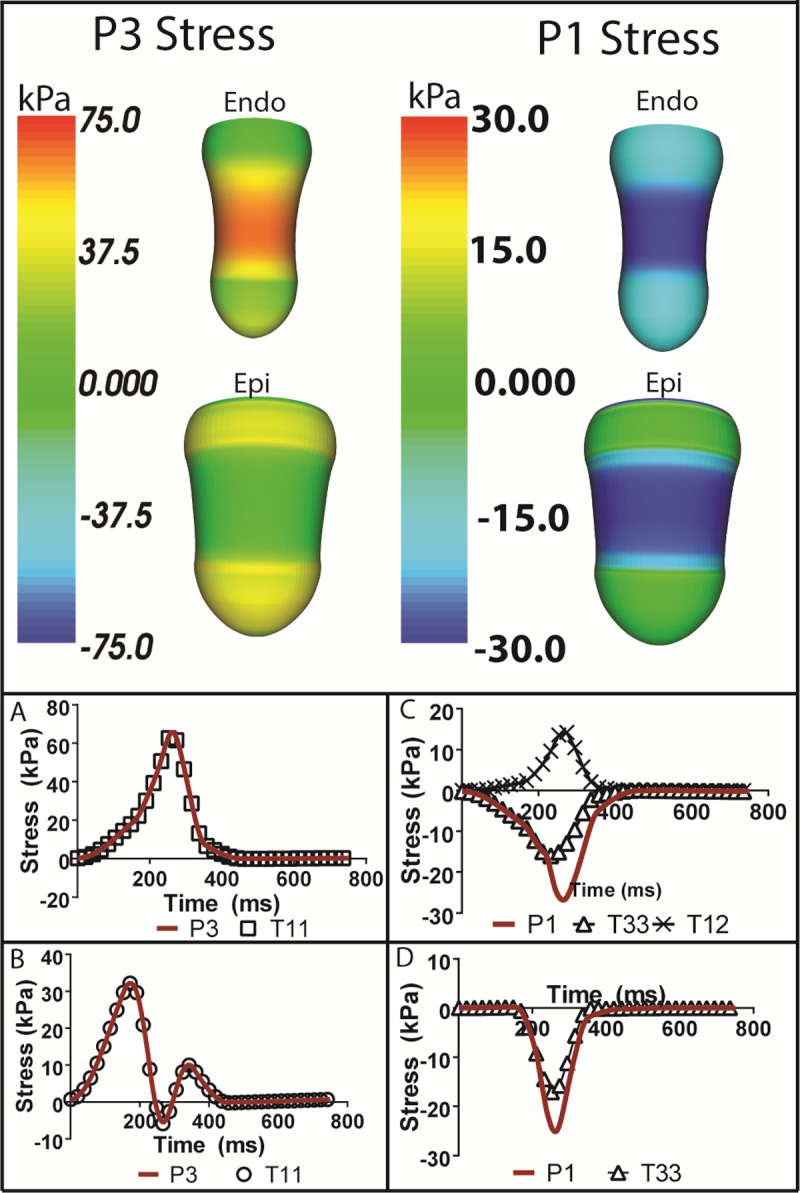
Principal stress rendering and time plots of principal and key contributing stress components in fiber coordinates. (Top) Maximum and minimum principal stress renderings on the epicardial and endocardial surfaces at peak compression. (A and B) Time series of maximum endocardial and epicardial principal stress and main contributing stress components. (C and D) Time series of minimum endocardial and epicardial principal stress and main contributing stress components. Note: All time renderings come from the middle of the compression. P3 is the maximum principal stress. P1 is the minimum principal stress. Fiber coordinates 1, 2 and 3 correspond to fiber, cross-fiber and transmural directions, respectively, i.e., T11, T22 and T33 are the normal stresses.

As shown in **Fig 8 (Top)** and **Table 3**, the maximum and minimum principal stresses are distributed over the endocardium beneath the compression zone and fall off rapidly toward the apex and base. In the epicardium, the maximum principal stress is smaller and is distributed outside of the compression zone while magnitudes are very small within the compression zone. The minimum principal stress is distributed over the compression zone and falls off rapidly toward the apex and base. The maximum principal stress at node B1a is 1.61 kPa in the baseline HF simulation. This increases with increased loads to moderate values, reaching a maximum value of 21.8 kPa at 1.5 NC. Maximum stress at node B1b is 46.2 kPa in the HF simulation. This value declines with increasing levels of peak loading, decreasing to 24.9 kPa at 1.5 NC. Maximum stresses at nodes C1a and C2a are very similar as a function of loading. They have maximum stresses of 45.8 and 44.2 kPa, respectively, in the HF simulation. They both decrease as maximum loads are increased. At NC, they have values of 32.2 and 32.1 kPa, respectively. Maximum stresses at nodes C1b and C2b have nearly identical values in the HF case. However, as maximum loads are increased, stresses at node C1b increase more than those at C2b. At NC they have values of 65.7 and 44.5 kPa, respectively. At 1.5 NC stresses are 145.0 and 65.7 kPa, respectively (see Discussion). The minimum principal stresses at nodes B1a and B1b increase as maximum load increases, i.e., they have larger absolute values and are more negative. Minimum stress at B1a has a large increase from -38.6 kPa at HF to -60.1 kPa at 1.5 NC. In contrast, the minimum stress at B1b increases much less as maximum load increases, changing from -12.7 kPa for the HF case to -17.2 kPa at 1.5 NC. The minimum stresses at the rest of the nodes (C1a, C1b, C1a, C2b) increase with increasing maximum loads. The minimum stresses at nodes C1a and C2a are negligible. As peak loading is increased to 1.5 NC, the minimum stress at C1a is larger than at C2a (-45.2 vs. -21.9 kPa, respectively). The minimum stresses at nodes C1b and C2b are identical at baseline with a value of -12.6 kPa, but values diverge as the maximum load increases. At 1.5 NC, the minimum stresses at C1b and C2b are -56.4 kPa -25.7 kPa respectively (see Discussion).

Time profiles of the maximum and minimum principal stresses in the endocardium and epicardium, as well as their major contributing components in fiber coordinates, are provided to show how they develop during the cardiac cycle. Looking at the maximum principal stresses **(Fig 8A and 8B)**, the fiber stress is the dominating factor for both the endocardium and the epicardium, respectively. The minimum principal stresses behave differently. In the endocardium **(Fig 8C)**, a portion of the minimum principal stress is composed of transmural compressive stress. However, there is significant fiber/cross fiber shear stress that is positive. In the epicardium **(Fig 8D)**, this is not as prevalent a factor, with the transmural compressive stress accounting for most of the minimum principal stress.

## Discussion

Multiscale simulation of cardiac compression using an axisymmetric cardiac mechanics model corresponding with initial compression sleeve prototypes indicates that large increases in left ventricular ejection fraction and stroke work can be obtained with moderate increases in myocardial wall strains and stresses. At some locations in the ventricular wall, key strain and stress components change little or even decrease with increasing levels of compression. Moreover, the hemodynamic results show that end-systolic volume decreases substantially with compression. If this result is born out in biventricular simulations and experiments, it could indicate that the proposed methods promote reverse remodeling in some cases. Comparison of key stress output from simulations with 9 and 17 applied force nodes indicates that longitudinal refinement as currently employed (17 nodes) may not be necessary in a future biventricular model. This feature may allow fewer circumferences in the large, more complex model and help reduce runtimes, as the differences between the left and right ventricles, need for nonuniform applied forces and change from single to dual closed-loop circulatory models will increase model size and runtime substantially.

The stresses estimated at the epicardial and endocardial nodes in the compression zone are distinctly different **(Fig 3)**. The epicardial nodes in the center of the compression zone have small negative, compressive stresses at peak compression. In contrast, the endocardial nodes have substantial positive, tensile stresses at this time in the cardiac cycle. This is a complex, three-dimensional deformation as shown in the strain time profiles **(Fig 7)**. The fiber architecture plays a large role since the myofibers are much stiffer in the fiber direction than the transverse plane. And, the variation in fiber direction is large with fibers oriented at -37 degrees from the circumference at the epicardium and +83 degrees or almost longitudinal at the endocardium. When fibers are loaded substantially, stresses will also be large. This is observed on the endocardium at peak compression. The ventricle is substantially elongated resulting in large, positive fiber strain on the endocardium and substantial associated fiber stress **(Fig 8)**. These components contribute greatly to the maximum principal strains and stresses. However, on the epicardium, modest stretching of the fibers occurs earlier in the cycle **(Fig 7)**, so that fiber stress and the associated maximum principal stress is much smaller by the time of peak compression. Moreover, it is apparent that substantial end effects occur toward the edges of the compression zone on the epicardium where shearing may also contribute.

The difference in curvature as well as the fact that the endocardium compresses inwards more than the epicardium may be due to the fiber architecture of the heart and the fact that the heart thickens during each contraction. An Australian group conducted an experiment on a sheep’s heart undergoing cardiac compression wherein a cup was placed around the ventricles and a pressure of 2.7 kPa was applied[20]. The authors measured an increase in the EF of the animal’s left ventricle from a baseline HF state of 21% to 33.5% with cardiac compression. It is promising that using similar pressures (3.1 kPa) on this idealized, single ventricle heart model yields increases in EF quite similar to these ovine measurements. However, this result needs to be validated in more realistic biventricular simulations and corresponding animal experiments.

The linear dependence of the hemodynamics on applied forces is promising since it would allow for tailoring the applied compression to the amount of cardiac support a failing heart might need. However, this is in a simplified geometric model, and this relationship will need to be explored in depth in proposed work using a biventricular model. Since the original biventricular model was constructed using clinical data from one patient, successful implementation of these methods in widely varying HF patients would likely require a precision medicine approach. It could be useful to perform patient-specific computational modeling to provide clinical decision support. However, it is premature to consider this potential use. Detailed results need to be obtained in which experimental data from a substantial number of animal studies are correlated with animal-specific computational models as a prelude to any proposed methods in the clinic. Such a combined experimental and computational approach will help elucidate the primary mechanisms of cardiac compression and the balance between improvements in hemodynamics and increases in either strains or stresses that might damage myocardial tissue or cause warping of a valve orifice.

The increase in the peak transmural thickening commensurate with peak compression suggests that applying an external load may augment the thickening mechanism of the heart. In these simulations, when applying epicardial forces of 7.5 N (3.1 kPa), the wall thickening increased from 8% to 13% **(Fig 6A)**. Although we can only hypothesize what the cause of the thickening increase is, an interesting possibility involves the change in circumferential or hoop stress with compression. The apex and base wall thickness time tracings **(Fig 6C and 6D)** are not as straightforward. Both have peak thinning around the time of maximum compression. But later in the cardiac cycle, thickening is as great or greater than baseline at these two locations, substantially greater at the apex. These results are difficult to interpret due to potential end effects and the boundary constraints at the base simulating the valve plane. However, the general increase in thickening might be attributed to the fact that compression decreases ESV and associated wall strains, which could allow for more myocardial contraction and thickening. In the current model, an asymmetric fiber architecture is used. The circumferential fibers are located about one third of the transmural depth from the epicardium. Therefore, fiber stress is identical to hoop stress at that depth throughout the model. We found that there is a substantial decrease in hoop stress as compression is applied **(Fig 6B**). The stress was lower than baseline HF model estimates throughout the cardiac cycle. While the baseline stress was tensile and monotonic, as would be expected, the stress was compressive and substantially so at peak compression. It is conceivable that this unloading of the circumferential fibers helps the heart thicken more than baseline. This phenomenon would need to be reproducible in more realistic biventricular simulations and experimentally, but it may suggest a synergy between baseline active state and applied compression.

Finite element nodes at the ventricular base are on the level of the valve plane. Attention must be paid to strains and stresses near these nodes and, in particular, adjacent to the low-pressure inflow valves. Results of this study indicate that maximum and minimum principal strains remain small there for all loading scenarios. Maximum principal stresses (tensile) are also small to moderate, but the minimum principal stress (compressive) at the basal epicardium rises to large values for compression greater than nominal loading and may serve to constrain the magnitude of compression.

Maximum principal stresses near the endocardium at the center and apical end of the compression zone are moderate at baseline but increase substantially with increased compressive loading. Values in the center increase more than those toward the apex, but they aren’t large for nominal compression. However, for loading 50% higher than nominal, the center value is very large and may provide a constraint on allowable peak loading. Conversely, maximum stresses decrease near the epicardium indicating an inverse relationship between inner and outer regions of the wall during compression. Minimal principal stresses in the compression zone are small to moderate at baseline and loading up to nominal values. However, they are substantial in the center of the zone for loading 50% greater than nominal at both epicardium and endocardium.

Maximum and minimum principal strains in the compression zone are small to moderate at baseline and for most compressive loading scenarios. Maximum values are moderately large only when loads are greater than nominal and may serve to limit compression. Minimum values become substantial only for loading greater than nominal near the endocardium at the center of the compression zone.

The complexity of the strains is predominantly due to the changing active state of the myocardial fibers and the interaction of applied compression with the underlying baseline contraction. Throughout the cardiac cycle, increased levels of calcium will cause a stiffening but also a contraction of the myocardial fibers and alter which strains in fiber coordinates dominate the principal strains. Looking at the maximum principal strains, various components are dominating at differing times during the cardiac cycle. In the epicardium, the maximum principal strain begins in the fiber direction, transitions to the transmural direction once the fiber stiffens, and transitions back to the fiber direction towards the end of the cardiac cycle and diastolic filling. The opposite occurs in the maximum principal endocardial strain, with the cross-fiber strain dominating initially until the fiber contracts, causing a shift to the fiber strain, with an eventual return to the cross-fiber strain during diastolic filling. The stresses are much simpler, with a contracted fiber being stiffest in the fiber direction, which is evident in the analysis of maximum principal stresses in terms of the component stresses in fiber coordinates.

There are three conclusions that can be drawn from the principal stresses and strains as well as their components in fiber coordinates: **(1)** The majority of the maximum principal stress will occur in the fiber direction since it is the stiffest direction relative to the transverse plane at any ventricular site. **(2)** The minimum principal stress will typically be compressive in the transmural direction since an external force or equivalent pressure is applied in that direction. And **(3)** The maximum principal strain exhibits considerable time dependence on which components in fiber coordinates dominate throughout the cardiac cycle.

Currently, LVADs and other mechanical circulatory support devices are typically reserved for NYHA Class 4 heart failure. If a synergism of applied compression and the baseline active state of the heart can be elucidated further and it relies on native cardiac function, it is possible that a healthier heart will either provide more synergism and associated wall thickening or require less applied force to restore cardiac function. This could lead to a larger patient cohort indicated for treatment using these methods.

Right now, the simulations do not account for reverse remodeling after implantation. However, in practice, when remodeling does occur, it is plausible that a new compression profile will need to be implemented depending on the extent of any remodeling that may occur. For very enlarged hearts as observed frequently in dilated cardiomyopathy, it is reasonable that under chronic cVAD use, the heart will reverse remodel to a smaller size. And a smaller sleeve will need to be used if assistance is still required to achieve adequate hemodynamics. Therefore, it may be useful for sleeve designs to incorporate the ability for adjustments in size, particularly circumference. This modified sleeve will likely need to apply less force since the heart will be healthier. Ideally chronic decreases in ESV will be observed in long term experimental studies and clinically, but this is beyond the scope of this initial study.

### Limitations

This type of cardiac compression may only benefit patients with dilated cardiomyopathy, about 50% of all cases. It does not necessarily apply to heart failure without dilation or to heart failure with normal ejection fractions. Although the single ventricle computational model used in the current study is designed to correspond with initial compression sleeve prototypes, there are several assumptions and limitations. The idealized geometry of the left ventricular model does not capture some of the complexities of a biventricular heart; it lacks the right ventricle and corresponding pulmonary circulatory system. The boundary conditions are considerably simplified compared with those typically applied in a biventricular model. And, a more realistic model is no longer axisymmetric. Nevertheless, virtually all of the other features of our typical multi-scale, patient-specific biventricular model is incorporated in the current LV model. The overall left ventricular size is taken from that of the same mid-range biventricular heart failure model used in studies of an applied torsion VAD[19]. In addition, the constitutive equation and dynamic model of active state (excitation-muscle contraction coupling) are identical to the biventricular heart failure model. Small changes to the single, closed-loop circulatory model in relation to its counterpart in the systemic circulation of the biventricular model had to be made to ensure rapid convergence of the LV model. But these simulations are not meant to establish substantial conclusions on the optimization of a cardiac compression sleeve for use with the heart, but rather to develop hypotheses about the cardiac mechanics underlying the hemodynamic and regional mechanics consequences of cardiac compression, as well as investigate some potential metrics to be studied in future parametric studies using more realistic biventricular models and corresponding animal experiments.

### Future directions

The immediate next step is to move this compression framework into a validated patient-specific biventricular model of severe heart failure. This will generate some design trends for the development of soft robotic cardiac compression sleeves beyond existing symmetric sleeve prototypes. Using this approach, custom force profiles can be applied to the biventricular models. This would allow for exploring variations in compression on the left and right ventricles as well as from base to apex. In addition, a series of animal experiments must be performed to validate findings from the left ventricular and biventricular simulations and to provide a starting point for patient-specific applications of this technology in the clinic.

## Supporting information

S1 Data (2A) Scaled sinusodial force across the entire cardiac cycle (750 ms). (2B Time) The time series for the simulation with a step size of 2 ms. (2B Volume) Left ventricular volume throughout the cardiac cycles for the heart failiure case. (2C Time) This is the same as 2B Time series. (2C Pressure) Left ventricular pressure thoughout the cardiac cycles for the heart failure case. (2D Volume) Left Ventricular volume for the converged cardaic cycle of the heart failure case. (2D Pressure) Left Ventriuclar pressure for the converged cardic cycle of the heart failure case. (3A 9 Node Number) Arbitrary indenfication of the epicardial nodes used in the stress analysis. (3A 9 Node Stress) Maximum principal stress values at each of the epicardila nodes nodes. (3A 17 Node Number) Same indentification of the epicardila nodes but for the more refined model. (3A 17 Node Stress) Same stress calculation at the nodes for the more refined model. All 3B parts are the same as their respecitve 4A parts but for the endocardium. (4B noVAD Volume) Left ventricular volume in the converged caridac cycle of the heart failure model. (4B noVAD Pressure) Left ventricular pressure in the converted cycle of the heart failure model. (4B cVAD Volume) Left ventricular volume in the converged caridac cycle of the compression model. (4B cVAD Pressure) Left ventricular pressure in the converted cycle of the compression model. (Fig 5A Force) Values of epicardial applied force normalized to the nominal force. (Fig 5A EF) The left ventricle ejection fraction of the converged cardiac simulation for each applied force. (Fig 5A Pressure) The left ventricle maximum pressure for the converged cardiac simulation for each applied force. (F5B Force) Same as Fig 5A Force. (F5B EDV) The end diastolic volume for the converged cardiac simulation for each applied force. (F5B ESV) The end systolic volume for the converged cardiac simulation for each applied force. (Time No Compression) The time series for the initial heart failure case. (Time Compression) The time series for the nominal compression case. (F6A No Compression Thickening) Thickening percentage of the myocardium in the middle of the compression zone for the HF case. (F6A Compression Thickening)Thickening percentage of the myocardium in the middle of the compression zone for the nominal compression case. (F6B No Compression Hoop Stress) Fiber stress at the transmural depth where fibers are oriented circumferentially in the heart failure case. (F6B Compression Hoop Stress) Fiber stress at the transmural depth where fibers are oriented circumferentially in the nominal compression case. (Fig 6C No Compression Thickening) Myocardial thickening at the apex of the heart in the heart failure case. (Fig 6C Compression Thickening) Myocardial thickening at the apex of the heart in the nominal compression case. (Fig 6D No Compression Thickening) Myocardial thickening at the base of the heart in the heart failure case. (Fig 6D Compression Thickening) Myocardial thickening at the base of the heart in the nominal compression case. (Time) Time series of the converged cardiac cycle for the nominal compression case. (F7A) Maximum strain in the endocardium. P3 –Maximum Principal Strain, E11 –Fiber strain, E22- Cross Fiber Strain, E33 –Transmural Strain. (F7B) Maximum strain in the epicardium. P3 –Maximum Principal Strain, E11 –Fiber Strain, E33 –Transmural Strain. (F7C) Minimum strain in the endocardium. P1 –Minimum Principal Strain, E22 –Cross Fiber Strain, E33 –Transmural Strain. (F7D) Minimum strain in the epicardium. P1 –Minimum Principal Strain, E33 –Transmural Strain, E12 –Fiber-cross fiber shear strain. (Time) Time series of the converged cardiac cycle for the nominal compression case. (F8A) Maximum stress in the endocardium. P3 –Maximum Principal Stress, T11 –Fiber Stress. (F8B) Maximum stress in the epicardium. P3 –Maximum Principal Stress, T11 –Fiber Stress. (F8C) Minimum stress in the endocardium. P1 –Minimum Principal Stress, T33 –Transmural Stress, T12 –Fiber–cross fiber shear stress. (F8D) Minimum stress in the epicardium. (P1) Minimum Principal Stress, T33 –Transmural Stress.
(XLSX)
